# Total Intravenous Anesthesia With Remimazolam and Remifentanil for Intraoperative Care of a Child With Venezuelan Heritage, at Risk for a Mitochondrial Disorder

**DOI:** 10.14740/jmc5358

**Published:** 2026-07-01

**Authors:** Michael Tobias, Kolapo Dairo, Natalie Slenkovich, Gregory Cambier, Joseph D. Tobias

**Affiliations:** a The Ohio State University, Columbus, OH, USA; b The Ohio State University College of Medicine, Columbus, OH, USA; c Department of Anesthesiology & Pain Medicine, Nationwide Children’s Hospital, Columbus, OH, USA; d Department of Anesthesiology & Pain Medicine, The Ohio State University College of Medicine, Columbus, OH, USA

**Keywords:** Mitochondrial myopathy, Oxidative phosphorylation, Venezuelan heritage

## Abstract

Recent clinical communications have alerted healthcare providers to potential concerns related to a previously undiagnosed disorder of mitochondrial function in children with maternal Venezuelan heritage. These patients have developed severe neurological damage following the administration of an apparently uneventful anesthetic that included the volatile anesthetic agent, sevoflurane. Subsequent analysis of family members and patients has identified a point mutation of the NADH dehydrogenase 4 (ND4) gene (mtND4 m.11232T>C), a subunit of complex I of the mitochondrial electron transport chain. Current recommendations include avoidance of volatile anesthetic agents and propofol. We present a 6-year-old child with Venezuelan heritage who presented for anesthetic care for reduction and percutaneous pinning of a supracondylar fracture. Total intravenous anesthesia (TIVA) was provided with remimazolam and remifentanil as the primary agents. Information regarding the current investigation of this novel mitochondrial defect is presented, options for anesthetic care reviewed, and the novel use of remimazolam and remifentanil as the primary agents for TIVA discussed.

## Introduction

In July 2025, the American Society of Anesthesiologists and the Society for Pediatric Anesthesia issued a joint communication to alert practitioners to reports from the Society of Anesthesiology of Chile, the Venezuelan Society of Anesthesiology, and the Colombian Society of Anesthesiology and Resuscitation, regarding severe neurological damage in five previously healthy children of Venezuelan heritage following the administration of general anesthesia for elective surgery [1]. The clinical manifestations included severe postoperative neurologic dysfunction with cerebral edema and progressive intracranial hypertension ultimately resulting in death in four of the five patients. Neuroimaging findings demonstrated bilateral basal ganglia involvement with additional involvement of the cerebellum, substantia nigra, and other deep gray matter structures, suggestive of hypoxic–ischemic encephalopathy.

Analysis of the family members and patients has identified a point mutation of the NADH dehydrogenase 4 (ND4) gene (mtND4 m.11232T>C), a subunit of complex I of the mitochondrial electron transport chain [2]. This work identified the defect as a single amino acid substitution (proline for leucine at position 158 of the ND4 subunit of respiratory complex I in the electron transport chain). *In vitro* studies showed that sevoflurane exposure in cells carrying this genetic variant resulted in a pronounced suppression of mitochondrial oxygen consumption, with prominent effects on complex I–dependent respiratory pathways. Previous work has demonstrated that complex I of the respiratory chain is a known target of both volatile anesthetic agents and propofol [3–6]. In the current cohort of patients, sevoflurane had been administered in all of the cases and was identified as the most likely etiologic agent. Given these concerns, alterations in the regimens used for general anesthesia have been suggested when patients with maternal Venezuelan heritage have been identified. We, for the first time, report the use of the novel benzodiazepine, remimazolam as the primary agent for TIVA in a child of Venezuelan heritage.

## Case Report

Review of this case and presentation in this format followed the guidelines of the Institutional Review Board of Nationwide Children’s Hospital (Columbus, Ohio). This review was conducted in compliance with the ethical standards of the responsible institution on human subjects as well as with the Helsinki Declaration.

The patient was a 6-year-old, 18.8 kg child with Venezuelan heritage who presented for reduction and percutaneous pinning of a supracondylar fracture. His past medical and surgery history were negative. Family history was negative for anesthetic complications. He had no allergies and was not currently on any medications. The patient had an intravenous (IV) cannula in place and was receiving maintenance IV fluids. He was held *nil per os* for 8 h, premedicated with IV midazolam (2 mg), and transported to the operating room where routine American Society of Anesthesiologists’ monitors were placed. Anesthesia was induced with remimazolam (5 mg administered as 2–2.5 mg boluses) and fentanyl (50 µg). Endotracheal intubation was facilitated by the administration of rocuronium (10 mg). Prevention against surgical site infectious was provided by cefazolin (1,000 mg). Maintenance anesthesia was provided by remifentanil starting at 0.3 µg/kg/min and remimazolam starting at 20 µg/kg/min. Depth of anesthesia was monitored by the bispectral index (BIS). An additional bolus dose of remimazolam was administered for a BIS in the high 70s. During surgical manipulation, the remifentanil infusion was increased to 0.5 µg/kg/min to control the hemodynamic response (increased heart rate). The surgical procedure lasted approximately 15 min. At the completion of the surgical procedure, the remifentanil and remimazolam infusions were discontinued, dexamethasone (4 mg) and ondansetron (2 mg) were administered for prophylaxis against postoperative nausea and vomiting, residual neuromuscular blockade was reversed with sugammadex (100 mg). The patient emerged from anesthesia in 10 min, his trachea was extubated, and he was transferred to the post-anesthesia care unit. The remainder of his postoperative course was unremarkable and he was discharged home.

## Discussion

The first reports of mitochondrial disorders were reported in the 1960s with anecdotal descriptions based on clinical findings, physical examination, muscle biopsy results, and biochemical criteria [7]. Lufts and colleagues at the Karolinska Institute in Sweden outlined the clinical course of a 35-year-old woman with a hypermetabolic state manifested as diaphoresis, weight loss, polydipsia, weakness, and polyphagia that was unrelated to thyroid dysfunction [8]. Further workup revealed morphological evidence of abnormal mitochondria in a muscle biopsy and biochemical documentation of uncoupling of oxidative phosphorylation in isolated muscle mitochondria. Subsequent to this, other reports appeared of various clinical constellations with abnormalities identified by histological examination of tissues and biochemical abnormalities linked to abnormal mitochondrial function. Subsequently additional disorders such as red ragged fibers and Kearns–Sayer syndrome were identified based on similar clinical, biochemical, and histologic findings with involvement of high energy dependent organ systems including skeletal muscle, the central nervous system, and the heart [7, 9]. The molecular era of mitochondrial disorders began in the 1980s with identification of specific cellular pathogenic processes and defects in the mtDNA (deletions, substitutions, or translocations) [10–12]. Continued work has resulted in the identification of approximately 300 pathogenic mutations of mtDNA demonstrating the significant heterogeneity of these disorders.

The heterogeneity of these disorders manifests not only through the variations in their mtDNA alterations, but also in the clinical expression and their response to general anesthetic agents [4, 6]. Concerns regarding patients with maternal Venezuelan heritage have uniformly been centered around volatile anesthetic agents, predominantly sevoflurane. Development of specific recommendations may be challenging as the initial reports of severe postoperative neurologic injury in patients of Venezuelan heritage were largely disseminated through personal communications and non–peer-reviewed sources, limiting early understanding of the underlying pathophysiology and evidence-based perioperative management. However, more recent investigations have identified a mitochondrial DNA mutation involving the ND4 subunit of complex I of the electron transport chain, placing this disorder within the general classification of a mitochondrial myopathy. The maternally inherited mutation appears to confer increased susceptibility to anesthetic-induced mitochondrial dysfunction, with affected individuals often lacking baseline clinical manifestations prior to anesthetic exposure. In reported cases, volatile anesthetic exposure, specifically sevoflurane, has been consistently associated with catastrophic neurologic outcomes, including basal ganglia injury, cerebral edema, and death.

Anesthetic management of patients with suspected mitochondrial dysfunction remains challenging and is guided largely by a general biochemical understanding and limited clinical data. Volatile anesthetic agents are generally avoided in this population due to their inhibitory effects on the mitochondrial respiratory chain, particularly complex I. These agents impair oxidative phosphorylation and reduce ATP production, which may precipitate metabolic failure in patients with underlying defects in mitochondrial energy metabolism. Propofol has also raised concern given its well-described effects on mitochondrial function with experimental and clinical data demonstrating that propofol interferes with electron transport chain activity, including inhibition of complex I, II, IV, and acylcarnitine transferase resulting in impairment of oxidative phosphorylation [4, 13, 14]. Patients with underlying mitochondrial disorders may be at increased risk of the inhibitory effects of propofol on mitochondrial function while propofol exposure has been reported to unmask previously undiagnosed mitochondrial disease. In the context of Venezuelan patients with suspected complex I defects, the safety of propofol remains uncertain. Although anecdotal reports suggest that a single bolus dose of propofol during the induction of anesthesia is without consequence, prolonged infusions are currently not recommended. Given these concerns, alternative anesthetic strategies have been proposed that minimize interference with mitochondrial function including benzodiazepines, dexmedetomidine, ketamine, and opioid-based regimens, which are considered to have more favorable mitochondrial profiles, and have not been implicated in postoperative sequelae in patients with mitochondrial disorders [15–17]. Alternatively, regional anesthesia with procedural sedation using benzodiazepines and opioids offers another option for selected surgical procedures [18]. This recommendation is included in a recent consensus statement from the European Society of Anesthesiology & Intensive Care and the European Society of Pediatric Anesthesia [19]. Their recommendations states that: “Whenever appropriate for the surgical procedure, regional anaesthesia techniques may be considered.”

Remimazolam is a novel, ultra–short-acting benzodiazepine that acts as a GABA_A_ receptor agonist, producing amnesia, sedation, and anxiolysis. Although not currently FDA-approved for use in pediatric patients, clinical experience has demonstrated its efficacy as a primary or adjunctive agent for sedation and general anesthesia in the pediatric population [20]. Remimazolam undergoes rapid metabolism by tissue esterases, resulting in a short context-sensitive half-life and predictable recovery profile. When compared with propofol, it has been shown to have a more favorable hemodynamic profile with lower rates of hypotension, supporting its use in vulnerable populations. Preliminary anecdotal clinical evidence has demonstrated its safety in patients with various mitochondrial disorders including MELAS (mitochondrial encephalomyopathy, lactic acidosis, and stroke-like episodes) as well as its beneficial physiologic effects on mitochondrial function [21]. When combined with remifentanil, the two agents have been successfully used for TIVA in various clinical scenarios. Remimazolam is compounded from a lyophilized powder (20 mg) with 0.9% sodium chloride, resulting in a solution with a final concentration of 2.5 mg/mL.

In our patient, the combination of remimazolam and remifentanil provided successful intraoperative anesthetic care during this brief surgical procedure. Anesthesia was induced with two bolus does of remimazolam (each of 2.5 mg) for a total dose of 0.25–0.3 mg/kg with fentanyl (2.5–3 µg/kg) and rocuronium to facilitate tracheal intubation. Maintenance anesthesia was provided by remifentanil starting at 0.3 µg/kg/min and remimazolam starting at 20 µg/kg/min. An additional bolus dose of remimazolam was administered for a high BIS level and the remifentanil infusion was increased to 0.5 µg/kg/min to control heart rate during surgical manipulation. BIS monitoring provided real-time assessment of anesthetic depth and guided dose adjustments of remimazolam to ensure the appropriate level of general anesthesia [22]. However, it is crucial to acknowledge the potential limitations of depth of anesthesia monitoring with the BIS when employing remimazolam, as the databases used to develop the BIS algorithm were based on electroencephalograph readings from patients receiving propofol and the volatile anesthetic agents, not benzodiazepines including remimazolam [23]. As such, it may be that propofol and the volatile anesthetic agents affect the BIS differently from remimazolam [24].

### Learning points

Recent alarming clinical outcomes have demonstrated the potential devastating consequences of general anesthesia in a small subset of patients with Venezuelan heritage related to alterations in mitochondrial function. To prevent these devastating perioperative complications, we have instituted routine screening of patients with Venezuelan heritage during our preoperative questionnaires. Until there is more information, we have modified our anesthetic care for all patients with maternal Venezuelan heritage, regardless of when they moved to the United States. This specific mitochondrial mutation may be most common in patients with maternal heritage from the Carabobo region of Venezuela. However, pending further information, we continue to include all patients with maternal Venezuelan heritage.

At present, routine population-wide genetic screening is not being universally recommended. However, testing may be considered in individuals with Venezuelan maternal ancestry undergoing planned surgical procedures when testing is accessible and feasible. In the absence of such testing, there is limited evidence-based medicine regarding the optimal intraoperative care for these patients. Currently, avoidance of the volatile anesthetic agents and propofol is recommended. We suggest that the novel benzodiazepine, remimazolam, in combination with remifentanil can be used successfully for TIVA in this challenging clinical scenario.

## Data Availability

Any inquiries regarding supporting data availability of this study should be directed to the corresponding author.
